# A Relationship between Carotenoid Accumulation and the Distribution of Species of the Fungus *Neurospora* in Spain

**DOI:** 10.1371/journal.pone.0033658

**Published:** 2012-03-20

**Authors:** Eva M. Luque, Gabriel Gutiérrez, Laura Navarro-Sampedro, María Olmedo, Julio Rodríguez-Romero, Carmen Ruger-Herreros, Víctor G. Tagua, Luis M. Corrochano

**Affiliations:** Departamento de Genética, Universidad de Sevilla, Sevilla, Spain; Pacific Northwest National Laboratory, United States of America

## Abstract

The ascomycete fungus *Neurospora* is present in many parts of the world, in particular in tropical and subtropical areas, where it is found growing on recently burned vegetation. We have sampled the *Neurospora* population across Spain. The sampling sites were located in the region of Galicia (northwestern corner of the Iberian peninsula), the province of Cáceres, the city of Seville, and the two major islands of the Canary Islands archipelago (Tenerife and Gran Canaria, west coast of Africa). The sites covered a latitude interval between 27.88° and 42.74°. We have identified wild-type strains of *N. discreta*, *N. tetrasperma*, *N. crassa*, and *N. sitophila* and the frequency of each species varied from site to site. It has been shown that after exposure to light *Neurospora* accumulates the orange carotenoid neurosporaxanthin, presumably for protection from UV radiation. We have found that each *Neurospora* species accumulates a different amount of carotenoids after exposure to light, but these differences did not correlate with the expression of the carotenogenic genes *al-1* or *al-2*. The accumulation of carotenoids in *Neurospora* shows a correlation with latitude, as *Neurospora* strains isolated from lower latitudes accumulate more carotenoids than strains isolated from higher latitudes. Since regions of low latitude receive high UV irradiation we propose that the increased carotenoid accumulation may protect *Neurospora* from high UV exposure. In support of this hypothesis, we have found that *N. crassa*, the species that accumulates more carotenoids, is more resistant to UV radiation than *N. discreta* or *N. tetrasperma*. The photoprotection provided by carotenoids and the capability to accumulate different amounts of carotenoids may be responsible, at least in part, for the distribution of *Neurospora* species that we have observed across a range of latitudes.

## Introduction

The ascomycete fungus *Neurospora crassa* is used as a model organism for research on different aspects of eukaryotic molecular biology including RNA inactivation and gene silencing, the mechanism of genetic recombination, regulation by the circadian clock, and the regulation by light of gene expression [1]–[4]. In addition, a collection of more than 4000 *Neurospora* wild-type strains from natural populations has provided information about the distribution of the different species of *Neurospora* in the world [5], [6]. Genomic analysis of wild-type strains of *Neurospora* has allowed detailed characterization of the process of adaptation to local environmental conditions [7]. Most of the *Neurospora* species have been identified in tropical or subtropical areas where they can be easily spotted growing on the surface of recently burned vegetation. Extensive surveys have extended the geographical distribution of *Neurospora* to temperate areas of western North America and Europe, with colonies of *N. discreta* identified as far north as Alaska [8], [9]. The distribution of *Neurospora* species in nature is varied. For example, *N. discreta* is the most frequent *Neurospora* species isolated in western North America [8] but in Europe *N. discreta* was rarely found while *N. crassa*, *N. sitophila*, and *N. tetrasperma* were frequently observed [9].

The colonies of *Neurospora* are very conspicuous due to the accumulation of the orange carotenoid neurosporaxanthin in conidia and vegetative mycelia [10]. The biosynthesis of neurosporaxanthin in vegetative mycelia is induced by light [11] through the activation of the biosynthetic genes [12], [13]. Light serves as an environmental cue to adjust the circadian clock so that *Neurospora* can anticipate changes in environmental conditions [12], [14]–[16]. Carotenoids, like neurosporaxanthin, are antioxidants due to their capacity to quench reactive oxygen species [17]–[19], and provide protection against UV damage in human skin [20] and fungi [21]–[23], but not against gamma-radiation in the fungus *Phycomyces blakesleeanus* [24]. Pigmented strains of the basidiomycetous yeast *Sporobolomyces ruberrimus* and *Cystofilobasidium capitatum* were more tolerant to UV damage and showed better survival after UV treatment than unpigmented strains [21]. The accumulation of carotenoids protected the yeast *Rhodotorula mucilaginosa* from UV damage [23]. In animals, a photoprotective role for carotenes in echinoids eggs has been suggested [25], and mice fed with beta-carotene or canthaxanthin were protected against skin tumors caused by UV radiation [26]. Carotenoids provide protection against excess irradiation in photosynthetic organisms. For example, accumulation of carotenoids (beta-carotene, zeaxanthin, or canthaxanthin) in strains of the cyanobacterium *Synechococcus* after transformation with carotenogenic genes led to protection of photosynthesis from UV damage [27], [28]. The activation by light of carotenoid biosynthesis in *Neurospora* and other fungi [29] may optimize protection against UV radiation when the fungus is growing exposed to light in open environments. Tropical regions (low latitude) receive more solar radiation than northern locations (high latitude). For a given site the amount of solar radiation changes during the time of the year, the altitude and the atmospheric conditions, but during the summer low-latitude locations receive twice the amount of UV-B radiation (280–315 nm) than high-latitude locations [30], [31]. It is possible that the capability to accumulate carotenoids may affect the distribution of *Neurospora* species in low-latitude areas due to a high exposure to UV radiation.

The region of Galicia (northwestern corner of the Iberian peninsula) and the two major islands of the Canary Islands archipelago (Tenerife and Gran Canaria, west coast of Africa) suffered an unusual number of wildfires during the summers of 2006 and 2007 respectively. Since *Neurospora* is easily spotted on burned vegetation, these summer fires allowed the opportunity to sample the *Neurospora* populations in these separated areas. We have observed differences in the distribution of *Neurospora* species in each site. The four *Neurospora* species that we have identified (*N. discreta*, *N. crassa*, *N. tetrasperma*, and *N. sitophila*) showed differences in the accumulation of carotenoids after light exposure. In addition, we have found a correlation between the accumulation of carotenoids in *Neurospora* and the latitude of the sampling site. Our results suggest that the capability to accumulate carotenoids plays a role in the distribution of *Neurospora* species in nature.

## Results and Discussion

### *Neurospora* in Spain

**Figure 1:**
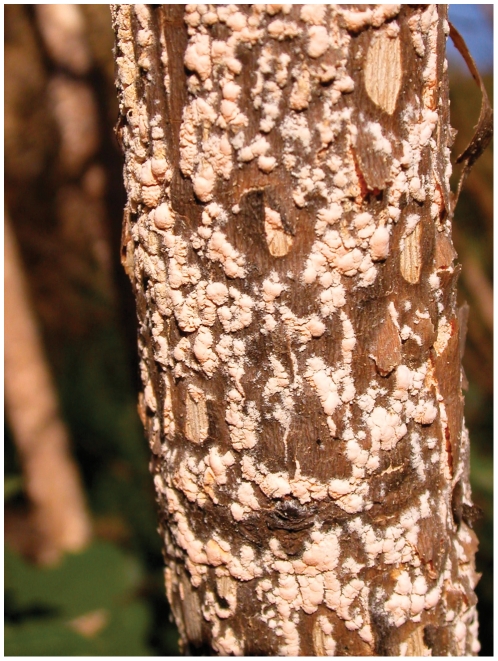
*Neurospora* growing on the trunk of a burned tree. Colonies of conidiating *Neurospora* are easily spotted by their orange color due to the accumulation of carotenoid pigments. The picture was taken in a garden in Seville (Spain) a few weeks after a summer fire in 2004 and is probably *Neurospora crassa* as all the samples taken from this site were later identified as belonging to this species.

Colonies of *Neurospora* were readily observed growing on the surface of partially burned vegetation (Fig. 1), as already observed previously in other European collection sites [9]. We collected *Neurospora* wild-type strains from eight sites located in the region of Galicia and from one site in the province of Cáceres (summer of 2006), and from six sites in the Canary Island archipelago (summer of 2007) that included samples from the two major islands (Tenerife and Gran Canaria). The sites were selected by their accessibility to locations where wild fires had occurred recently (4–6 weeks). The sites that we selected may not be representatives of the entire region. The location of the collection sites had latitudes that ranged from 27.88°N (Fataga and Morán, Las Palmas) to 42.74°N (Herbón, A Coruña). In 41 plants we took more than one sample in order to investigate the presence of genetic variations in the *Neurospora* strains that colonized a single plant. The samples were collected, purified from single colonies, and stored prior to further characterization. From the samples that we isolated in the field trips we purified and stored a total of 125 wild-type strains (Table S1). Our collection also includes 26 *Neurospora crassa* strains isolated from Seville in 2004 that have been reported previously [9].

### Identification of *Neurospora* species

**Figure 2:**
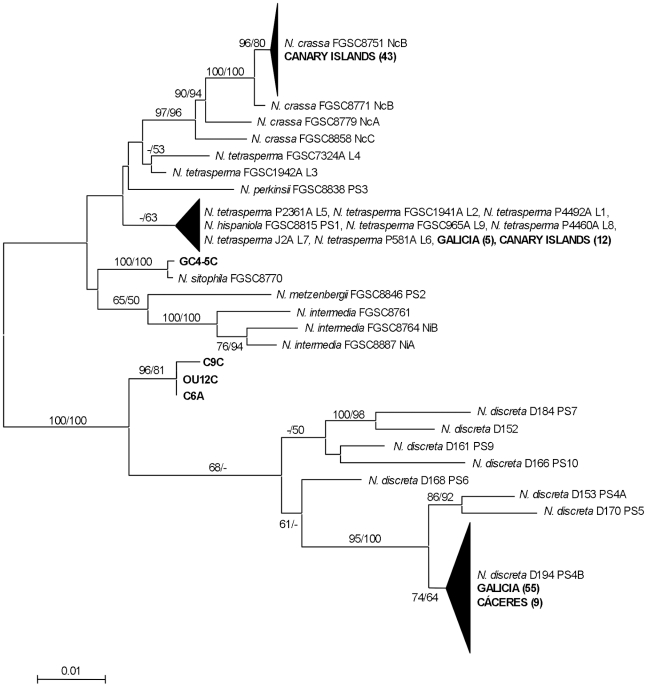
Identification of *Neurospora* species by phylogeny. Maximum-Likelihood tree produced from the TMI, DMG and TML loci combined. Branch support values (Maximum-likelihood bootstrap proportions/Neighbor-Joining bootstrap proportions) in combined analyses are displayed for major branches only. The well-supported groups of individuals are indicated by triangles, with height proportional to number of individuals and width proportional to the mean number of changes from the node. Only bootstrap proportions greater than 50% are shown. The number of isolates in shown in parenthesis.

We used a phylogenetic method to identify the species of each collected *Neurospora* strain. We amplified and sequenced three unlinked polymorphic loci (TMI, TML, and DMG) from each wild-type strain [32]. These loci have been used successfully to infer the evolution and diversity of species of the genus *Neurospora* [32]–[35]. The sequences from the three loci from each strain were combined and aligned with homologous sequences from a set of reference *Neurospora* strains [32], [35]. The resulting DNA alignment was used to reconstruct a phylogenetic tree by the Maximum-Likelihood and Neighbor-Joining methods (Fig. 2). The *Neurospora* species for each isolate was deduced by the presence of a reference *Neurospora* species close to each unknown strain in the phylogenetic tree. A group of isolates formed a clade with different *N. crassa* reference strains and were assigned to *N. crassa*. The Spanish *N. crassa* formed a clade with a reference *N. crassa* strain from clade B as previous European *N. crassa* isolates [9]. Similarly, a group of isolates formed a clade with reference *N. discreta* strains and were assigned as *N. discreta*. Finally, one strain (GC4-5C) formed a clade with *N. sitophila* and was assigned as a *N. sitophila* (Fig. 2).

Some strains could not be assigned using the phylogenetic method. Three isolates (C9C, OU12C, and C6A) formed a well-defined clade that separated before the major clade that included all the *N. discreta* reference strains (Fig. 2). These strains were classified as *N. discreta* but they may represent a new phylogenetic species. In addition, a group of strains formed a clade that included *N. tetrasperma*, and *N. hispaniola* (Fig. 2). As an alternative method to identify the *Neurospora* species corresponding to these strains we performed blast searches using DNA sequences for the TMI, TML, and DMG loci from each strain. Searches of the *Neurospora* DNA database with sequences from DMG and TML from strains C9C, OU12C, and C6A identified *N. discreta* DNAs as the most similar sequences, thus confirming these strains as *N. discreta*. Searches using TMI identified a variety of different *Neurospora* species and were not considered. Searches using the three loci from the group of putative *N. tetrasperma* strains were not conclusive as they identified a variety of different *Neurospora* species. We therefore amplified and sequenced a fourth locus (QMA) [32] from these strains. Blast searches using QMA sequences from these strains identified *N. tetrasperma* DNA in the *Neurospora* DNA database and supported the assignment of these strains as *N. tetrasperma*. In addition, these strains produced *Neurospora* sexual structures, perithecia, when grown in independent cultures. Matured perithecia contained asci with four ascospores, and the strains developed asexual conidia (not shown) and accumulated carotenoids (see below). These biological properties are specific for *N. tetrasperma*, a self fertile species that can reproduce in isolation due to the presence of nuclei with either mating type in the same hyphae (pseudohomothalism) [5]. The biological properties of these strains, and the similarities of their QMA locus with *N. tetrasperma* DNA supported our assignment of these strains as *N. tetrasperma*. Further characterization of the *Neurospora* wild-type strains by phylogenetic species recognition (PSR) [36] confirmed the *Neurospora* species assigned to each strain, with the exception of the *N. tetrasperma* strains.

### Distribution of *Neurospora* species in Spain

**Figure 3:**
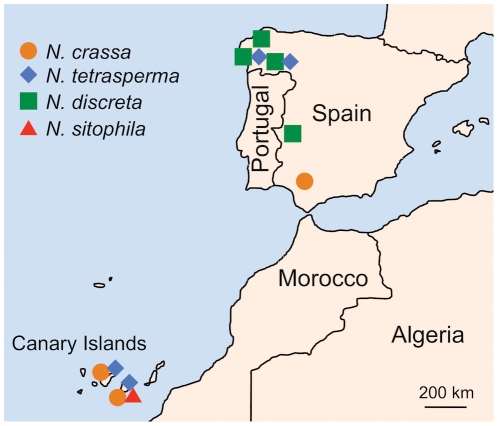
Distribution of *Neurospora* species collected in Spain.

We have identified 64 *N. discreta* strains, 17 *N. tetrasperma* strains, 69 *N. crassa* strains, and one *N. sitophila* strain (Table 1, Table S1). These heterothallic and pseudohomothallic species appear as a terminal clade in the phylogeny of the genus *Neurospora* [37]. The distribution of *Neurospora* species across the sites that we surveyed was varied, perhaps reflecting differences in humidity, altitude, and light exposure (Fig. 3). *N. discreta* was only found in Galicia and Cáceres, but was absent from southern sites. On the contrary, *N. crassa* was found in Seville and the Canary Island sites, but not in the northern sites that we sampled. *N. tetrasperma* showed a wide distribution as it was identified in Galicia and the Canary Islands (Fig. 3).

**Table 1:** Distribution of species of *Neurospora* across sites in Spain.

Province	Site	Latitude	Longitude	Altitude^a^	*N. discreta*	*N. tetrasperma*	*N. crassa*	*N. sitophila*	Total
A Coruña	Herbón	42.74°	−8.62°	79	16				16
Pontevedra	Cotobad	42.46°	−8.46°	444		1			1
	Lagoas	42.46°	−8.50°	344	3	1			4
	O Grove	42.42°	−8.84°	94	3	1			4
Ourense	Rouzós	42.42°	−7.92°	457	9				9
	Liñares	42.40°	−7.92°	407	6	2			8
	A Gudiña	42.05°	−7.12°	1075	11				11
	Lamas	41.98°	−7.54°	709	7				7
Cáceres	Cañaveral	39.80°	−6.38°	381	9				9
Sevilla^b^	Sevilla	37.37°	−5.99°	8			26		26
S. C. de Tenerife	La Guancha	28.37°	−16.65°	504		1	10		11
	Los Realejos	28.36°	−16.58°	737			3		3
	Masca	28.30°	−16.83°	1029		1	3		4
	S. del Teide	28.29°	−16.81°	908			2		2
Las Palmas	Mogán	27.88°	−15.72°	332			3		3
	Fataga	27.88°	−15.56°	638		10	22	1	33
*Total*					64	17	69	1	151

a Meters.

b The identification of these strains has been reported previously [9].

In 10 out of the 16 sites surveyed we found a single *Neurospora* species, the other six sites had either *N. discreta* or *N. crassa* and *N. tetrasperma* (*N. sitophila* was isolated in Fataga, a site where we also found *N. crassa* and *N. tetrasperma*). Previous characterization of *Neurospora* in Europe allowed the identification of a single species in only four out of 14 sites, therefore most sites had multiple *Neurospora* species [9]. Our discovery of a single isolate of *N. sitophila* in the Canary island of Gran Canaria was unusual, as several *N. sitophila* strains have been isolated in Spain and Portugal, and in other parts of Europe. *N. sitophila* represented 34% of all the *Neurospora* isolates in a European survey [9], and its nearly absence in our survey suggests that *N. sitophila* may have a restricted habitat that was not included in our field trips.

Most of the 41 plants where we isolated multiple independent samples were colonized by a single *Neurospora* species. In four cases we isolated two different *Neurospora* species growing in the same plant (three plants had *N. crassa* and *N. tetrasperma*, and one plant had *N. crassa* and *N. sitophila*). In six additional cases we found plants colonized by genetically different individuals (haplotypes) of *N. discreta*, resulting in a total of 10 out of 41 plants colonized with more than one *Neurospora* individual. This value is likely an underestimate of the frequency of plants colonized by different *Neurospora* individuals as we sequenced a very minor part of the genome, and we arbitrarily defined genetically different individuals as those that had more than 5% differences in the sequence of their three polymorphic loci (TMI, TML, and DMG). Our results show that more than one *Neurospora* individual can colonize and complete a vegetative cycle in a small area (a single plant) supporting previous observations [9], [38].

### Accumulation of carotenoids in *Neurospora* species

During the process of isolation and characterization of the *Neurospora* wild-type strains we noticed differences in the amount of carotenoids accumulated. We then assayed the amount of carotenoids accumulated by mycelia from each *Neurospora* strain after exposure to light during one day as compared to the accumulation observed in mycelia kept in the dark. We observed that all the strains had traces of carotenoids in mycelia kept in the dark (3–6 µg/g dry mass) but they differed in the amount of carotenoids accumulated after light exposure, and this difference was specific for each *Neurospora* species (Table 2, Table S1). *N. discreta* was the species that accumulated less carotenoids after light exposure (22.7 µg/g dry mass), while *N. tetrasperma* (100.9 µg/g dry mass) and *N. crassa* (141.1 µg/g dry mass) showed higher accumulations of carotenoids. The only *N. sitophila* strain that we isolated accumulated 142.7 µg/g dry mass of carotenoids. For a comparison the *N. crassa* standard wild-type strain (74-OR23-1VA) accumulated 225.2 µg/g dry mass of carotenoids (average of two experiments). Our results show that the *Neurospora* species that we have tested differed in their capabilities to accumulate carotenoids after light exposure.

**Table 2:** Accumulation of carotenoids in species of *Neurospora* isolated from Spain.

		Carotenoids^a^		Number of strains
	Latitude	Light	Dark	
*N. discreta*	42.74°-39.80°	22.7±1.5	3.9±0.2	63
*N. tetrasperma*	42.46°-27.88°	100.9±8.4	4.9±0.5	17
*N. crassa*	37.37°-27.88°	141.1±5.2	5.4±0.2	69
*N. sitophila*	27.88°	142.7±0.0	6.7±0.0	1
*N. discreta* Galicia	42.74°-41.98°	23.4±1.7	4.1±0.2	54
*N. discreta* Cáceres	39.80°	18.1±1.0	3.0±0.2	9
*N. tetrasperma* Galicia	42.46°-42.40°	66.7±3.8	3.1±0.3	5
*N. tetrasperma* Canary Islands	28.37°-27.88°	115.2±8.9	5.6±0.6	12
*N. crassa* Seville	37.37°	108.6±5.5	6.0±0.5	26
*N. crassa* Canary Islands	28.37°-27.88°	160.7±5.9	5.0±0.2	43

a Average±SEM (µg/g dry mass).

**Figure 4:**
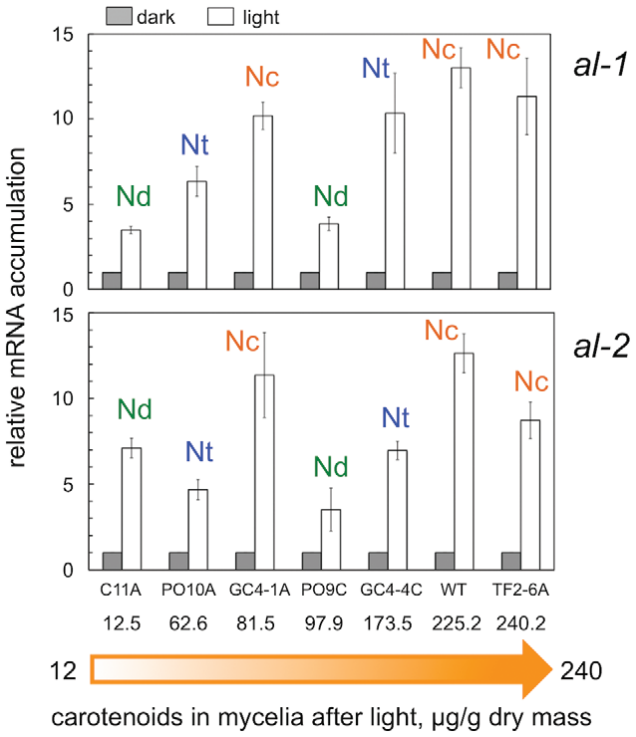
Activation by light of the *albino* genes in *Neurospora* species. Quantitative RT-PCR experiments were performed to measure the relative accumulation of *al-1* or *al-2* mRNA in mycelia of wild-type strains exposed to white light (2 W/m^2^ blue light) or kept in the dark. The plots show the average and standard error of the mean of the relative mRNA accumulation in four independent experiments, each with three replicates. The results from each PCR for each gene were normalized to the corresponding PCR for *tub-2* to correct for sampling errors and normalized to the result obtained with mycelia kept in the dark. The amount of carotenoids accumulated by each wild-type strain in cultures exposed to light is shown under each strain name. The initials describe each *Neurospora* species: Nc *Neurospora crassa*, Nt *Neurospora tetrasperma*, and Nd *Neurospora discreta*.

The differences in the accumulation of carotenoids by the species of *Neurospora* did not correlate with the expression of genes *al-1* or *al-2* (Fig. 4). These genes encode enzymes required for the biosynthesis of neurosporaxanthin. Gene *al-1* encodes the phytoene dehydrogenase [39], and *al-2* is a bifunctional gene responsible for the phytoene synthase and the carotene cyclase [40], [41]. The expression of these two genes is induced by light and we observed similar levels of light-dependent mRNA accumulation in strains that accumulate low or high amounts of carotenoids after exposure to light (Fig. 4). For example, the light-dependent *al-2* mRNA accumulation in strain C11A (*N. discreta*) that accumulated 12.5 µg/g dry mass of carotenoids was similar to the relative *al-2* mRNA amount observed in strain TF2-6A (*N. crassa*) that accumulated 240.2 µg/g dry mass of carotenoids. Similarly, we did not observe changes in the light-dependent *al-1* mRNA accumulation between strains C11A and PO9C (*N. discreta*) despite the ten-fold difference in carotenoid accumulation (Fig. 4). These results indicate that changes in gene expression, at least for *al-1* and *al-2*, are not responsible for the differences in the final amount of carotenoids accumulated after exposure to light that we have observed in different species of *Neurospora*. It is possible that alternative mechanisms, the activation by light of other key genes or the light-dependent modification of regulatory proteins, play a major role in the regulation of carotenoid accumulation in *Neurospora*.

### The accumulation of carotenoids in *Neurospora* shows a correlation with latitude

The amount of carotenoids that *N. discreta* strains accumulate after light exposure varied little regardless of their isolation site. However, we noticed that *N. tetrasperma* and *N. crassa* strains isolated in different locations differed in the amount of carotenoids accumulated after exposure to light (Table 2, Table S1). For example, the *N. tetrasperma* strains isolated in Galicia accumulated 66.7 µg/g dry mass in average compared to the 115.2 µg/g dry mass accumulated by the *N. tetrasperma* strains isolated in the Canary Islands (Table 2). Similarly, the *N. crassa* strains isolated in Seville accumulated 108.6 µg/g dry mass in average while the *N. crassa* strains isolated in the Canary Islands accumulated 160.7 µg/g dry mass in average (Table 2). These results show that the *Neurospora* strains isolated from lower latitudes (the Canary Islands) had the capability to accumulate more carotenoids after exposure to light than the *Neurospora* strains isolated from higher latitudes (Galicia and Seville).

**Figure 5:**
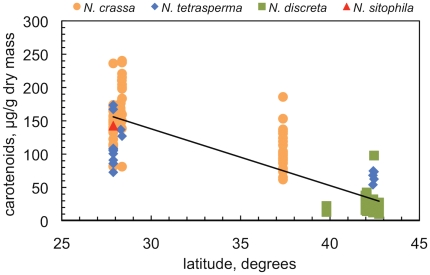
A negative correlation between carotenoid accumulation and latitude. Mycelia from each wild-type strain were exposed to light during one day and the amount of carotenoids measured. Each point represents the average amount of carotenoids obtained in two independent experiments and the latitude of the site where the wild-type strain was collected. The line shows the lineal regression for the 150 wild-type strains characterized (coefficient of determination R^2^ = 0.71).

In order to explore in more detail the relationship between carotenoid accumulation and the location of the isolation site we plotted the amount of carotenoids accumulated after exposure to light and the latitude of the collection site for the 150 wild-type strains that we have isolated (Fig. 5). The plot shows a negative correlation between carotenoid accumulation and latitude (coefficient of determination R^2^ = 0.71) and confirms the observation that *Neurospora* strains isolated from lower latitudes accumulate more carotenoids after exposure to light than strains isolated from higher latitudes. A two-way analysis of variance (ANOVA) between *Neurospora* species, latitude, and carotenoid accumulation confirmed that *Neurospora* species showed significant differences in carotenoid accumulation, and that the species isolated from different latitudes showed significant differences in carotenoid accumulation (p<0.001) (Table S2).

The negative correlation shown in Fig. 5 can be explained by the combination of two effects. First, we only detected *N. discreta* strains in high latitude sites, and these strains accumulated less carotenoids than *N. tetrasperma* and *N. crassa* (Table 2). *N. crassa*, the species with the highest accumulation of carotenoids, was only detected in low-latitude sites. Second, *N. tetrasperma* and *N. crassa* strains isolated in low-latitude sites accumulated more carotenoids after exposure to light than strains of the same species isolated from high-latitude sites (Table 2).

The sites that we have surveyed differed in many characteristics in addition to latitude, including altitude, average temperature, and humidity. We did not detect any relationship between altitude and carotenoid accumulation (not shown), but other environmental factors and differences in vegetation may have contributed to the correlation that we have uncovered.

There is a correlation between solar radiation and latitude as areas of low latitude receive more UV radiation than areas of high latitude [30], [31]. UV radiation damages DNA by inducing the formation of cyclobutane-pyrimidine dimers and 6-4 photoproducts [42], [43], and cells have molecular mechanisms to protect DNA from UV-induced damage [43]. In fungi the harmful effects of UV radiation are reduced after the activation by light of the biosynthesis of screening and photoprotective pigments, like carotenoids, and the activation by light of genes for DNA repair [11], [29], [44]–[47]. As UV irradiation is higher in regions of low latitude than in high-latitude locations we propose that the increased carotenoid accumulation that we have observed in *Neurospora* strains isolated from lower latitudes may provide an increased protection from high UV exposure. The photoprotection provided by carotenoids and the capability to accumulate different amounts of carotenoids may be, at least in part, responsible for the distribution of *Neurospora* species that we have observed in several areas of Spain. An alternative hypothesis would be that other unknown factors played major roles in the distribution of *Neurospora* species in nature and that the species increased their carotenoid accumulation during the process of adaptation to low latitudes while carotenoid accumulation remained less relevant in species adapted to high-latitude locations.

### Conidia of *N. crassa* are more resistant to UV radiation than conidia from *N. discreta* or *N. tetrasperma*

**Figure 6:**
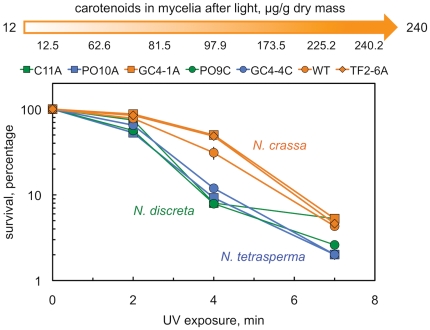
Survival of *Neurospora* conidia to UV radiation. Conidia were exposed to UV radiation during different times or kept unirradiated as a control. Cell viability was assayed after plating irradiated and unirradiated conidia, counting the number of colonies after 2–3 days of growth, and comparing the number of colonies obtained with irradiated and unirradiated conidia. The plot shows the average and standard error of the mean of the percentage of survival to UV in three independent experiments. The amount of carotenoids accumulated by mycelia from each wild-type strain in cultures exposed to light is shown over each strain name. The color used for symbols and lines identify each *Neurospora* species. The latitudes and longitudes of the isolation sites for each strain are the following: C11A (42.74°, −8.62°), PO10A (42.46°, −8.50°), GC4-1A (27.88°, −15.56°), PO9C (42.46°, −8.50°), GC4-4C (27.88°, −15.56°), TF2-6A (28.37°, −16.65°).

In order to explore in more detail the role of carotenoids in UV protection we assayed the survival of conidia from several *Neurospora* species after UV exposure (Fig. 6). To perform the assay we selected strains that accumulated low or high amount of carotenoids and the standard wild-type strain for a comparison. The capability to accumulate carotenoids in mycelia after exposure to light did not provide additional protection to UV exposure in our assay. For example strain GC4-1A (*N. crassa*) showed better survival after UV exposure than strain GC4-4C (*N. tetrasperma*), but GC4-1A accumulated less carotenoids than GC4-4C after exposure to light (Fig. 6). It should be noted that this assay was performed with conidia, and *Neurospora* conidia accumulate carotenoids constitutively due to the expression of the *al* genes [48], [49]. It is possible that the capability to accumulate carotenoids after exposure to light is more important for the survival of vegetative mycelia after UV irradiation than for the survival of conidia. The three wild-type strains of *N. crassa* that we assayed showed better survival after UV exposure than strains of *N. tetrasperma* or *N. discreta*, in particular after four min of UV exposure (Fig. 6). This could be due to the presence of high amounts of carotenoids in conidia, but other alternatives like an improved machinery for DNA repair cannot be ruled out. The greater resistance of *N. crassa* conidia to UV irradiation, the predominance of this species at collection sites at lower latitudes, and their ability to produce the most carotenoids, support the hypothesis that the distribution of *Neurospora* species across latitudes is influenced by light-induced carotenoid production.

A relationship between resistance to UV exposure and the latitude of the isolation site has been shown for the entomopathogenic fungi *Beauveria bassiana* and *Metarhizium anisopliae* as strains isolated close to the equator showed improved resistance to UV than strains isolated at higher latitudes [50], [51]. These observations suggest that resistance to UV radiation play a role in the distribution of fungi in nature, at least for the small number of examples investigated.

*N. crassa*, *N. discreta*, and *N. tetrasperma* have been isolated in additional locations across Europe [9], and *N. discreta* has been isolated as far north as Alaska [8]. Many *Neurospora* wild-type strains have been collected in locations around the world [6] but their carotenoid accumulation or their resistance to UV radiation have not been characterized. A comprehensive investigation of the relationship between carotenoid accumulation, UV resistance, and latitude will require extensive sampling of the *Neurospora* populations in different locations. These experiments will help to assess if the correlation between carotenoid accumulation and latitude that we have uncovered for the *Neurospora* populations in some areas of Spain are observed in a more global scale.

## Materials and Methods

### Ethics statement

No specific permits were required for this field study. In addition, no specific permission was required as the samples were taken from public areas and the study did not involve an endangered or protected species.

### Collection and culturing of *Neurospora* wild types

We made two field trips to fire sites located in opposites ends of Spain that had suffered an unusual high frequency of fires during the summers of 2006 and 2007. The first trip took place during October 2006 to visit Galicia at the northwestern corner of the Iberian Peninsula. The second trip took place during September of 2007 to visit the Canary Islands at the northwestern coast of Africa. Additional samples were taken in the province of Cáceres while travelling to Galicia. Most of the sites were surveyed 4–6 weeks after the end of the fire. All the samples were taken from colonies growing on the surface of burned vegetation but we did not pay attention to the orientation relative to the sun of the sampling site. The location of each sampling site is described in Table 1.

We followed standard methods of handling wild type isolates of *Neurospora*, including collecting, initial culturing, subculturing of single conidia, and storage [8]. A field sample of conidia was collected from a sporulating colony onto sterile filter paper, which then was placed in a sterile envelope. One colony per plant was sampled for up to 33 isolates per site. In addition, where possible, up to four isolates from the same plant were collected per site. All the wild type strains have been deposited in the Fungal Genetic Stock Center (FGSC; http://www.fgsc.net) under accession numbers 10264–10289, 10461–10585 (Table S1).

We used the standard *Neurospora crassa* wild-type strain 74-OR23-1VA (FGSC 2489 *mat A*). All strains were maintained by growth in Vogel's minimal media with 1.5% sucrose as carbon source. Strain manipulation and growth media preparation followed standard procedures and protocols [3]. See also, the *Neurospora* protocol guide (http://www.fgsc.net/Neurospora/NeurosporaProtocolGuide.htm).

### Identification of *Neurospora* species by DNA sequence comparisons

Total genomic DNA was isolated from each wild-type strain and three polymorphic regions (unlinked, noncoding loci that flank microsatellites named TMI, TML, and DMG) were amplified by PCR, purified, and sequenced. We amplified and sequenced an additional polymorphic region (QMA) from the *N. tetrasperma* strains. The PCR conditions and the sequence of primers for the amplification of each polymorphic sequence have been described previously [32]. The DNA sequences have been deposited in GenBank with the following accession numbers: JN017932–JN018056 (DMG), JN030769–JN030893 (TMI), JN048514–JN048638 (TML), and JN084107–JN084123 (QMA).

The sequences of the three loci (TMI, TML, and DMG) from the wild-type strains were combined into a single dataset for phylogenetic analysis. Microsatellite sequences were omitted from the analyses. In the alignment we included the DNA sequences from the three loci obtained from a set of reference *Neurospora* strains that included *N. crassa*, *N. discreta*, *N. tetrasperma*, *N. sitophila*, *N. intermedia*, and the recently identified *N. hispaniola*, *N. metzenbergii*, and *N. perkinsii* [32]–[36]. The sequences for each loci were aligned using Clustal X [52]. Phylogenetic analyses were performed using MEGA4 [53]. Phylogenetic reconstruction was applied to the three loci independently and their combined alignment. The evolutionary history was inferred using the Maximum Likelihood and Neighbor-Joining methods, reliability of the branches was estimated using the bootstrap method (500 replicates). The best nucleotide substitution model was inferred using jModelTest [54].

We identified the species of each *Neurospora* wild-type strain using three methods. The first method relied on the establishment of evolutionary relationships between the DNA sequences obtained from each wild-type strain and DNA sequences obtained from representatives of various *Neurospora* species based on the combined analysis of the three loci. Identification of *Neurospora* species using this multilocus genealogical approach has proven successful [32]–[35].

The second method to identify each wild-type strain was to search the *Neurospora* DNA sequences in GenBank for sequences similar to the three loci that we characterized from each wild-type strain (TMI, TML, and DMG) using the program Blast [55] accessed through the NCBI web server (http://www.ncbi.nlm.nih.gov/). We used default parameters of the Megablast algorithm that is optimized for highly similar sequences. The *Neurospora* species that gave the best hit after each Megablast search confirmed the species assignment obtained by phylogenetic comparisons. An additional loci (QMA) was used to identify *N. tetrasperma* strains using Megablast.

The third method is based on the Phylogenetic Species Recognition (PSR) concept described in [36]. A phylogenetic species is recognized if it satisfied either of two criteria: (1) Genealogical concordance: the clade was present in the majority (2/3) of the single-locus Maximum-Likelihood genealogies, as revealed by a majority-rule consensus tree. (2) Genealogical nondiscordance: the clade was well supported in at least one single-locus Maximum-Likelihood genealogy, as judged by bootstrap proportions and was not contradicted in any other single-locus genealogy at the same level of support. To identify such clades, a tree possessing only branches that received a bootstrap proportion >70% was chosen to represent each of the three loci, then a semistrict consensus tree was produced from these three trees. The semistrict tree was constructed with the program Component [56].

To identify genetic variations between each *Neurospora* species and among strains isolated from a single plant DNA sequences from each locus with a similarity higher than 95% were grouped using the program UCLUST with default parameters [57].

### Carotenoid analysis

Approximately 10^6^–10^7^ conidia were inoculated into 25 ml of Vogel's liquid minimal medium with 0.2% Tween 80 as wetting agent, and placed in sterile Petri dishes. The plates were grown in the dark for one day at 34°C and then exposed to light under a set of fluorescent bulbs (2 W/m^2^ blue light) at 22°C for one day or kept in the dark as a control. Mycelia were collected, frozen in liquid nitrogen, and lyophilized. Carotenoids were extracted from 0.1 g dry weight samples as described [40]. Total carotenoids were estimated from measurements of the maximal absorption spectra in hexane, assuming an average maximal E (1 mg/l, 1 cm) = 200.

### RNA purification and quantitative RT-PCR

Approximately 10^5^ conidia were inoculated into 25 ml of Vogel's liquid minimal medium with 0.2% Tween 80 as wetting agent, and placed in sterile Petri dishes. The plates were incubated in the dark for two days at 22°C and then exposed to light under a set of fluorescent bulbs (2 W/m^2^ blue light) at 22°C for 30 min or kept in the dark as a control. Mycelia were collected, frozen in liquid nitrogen, and stored at −80°C. For RNA extraction mycelia were disrupted by two 0.5-min pulses in a cell homogenizer (FastPrep-24, MP Biomedicals), in RNA extraction buffer, with 1.5 g of zirconium beads (0.5 mm diameter) in 1.9-ml screw-cap tubes. The samples were cooled on ice for 5 min after the first pulse. The extracts in screw-cap tubes were clarified by centrifugation in a microcentrifuge (13,000 rpm) for 5 min prior to RNA purification. Total RNA from mycelia was obtained using the RNeasy Plant Mini Kit (Qiagen). Quantitative PCR experiments were performed to determine relative mRNA abundance using one-step RT-PCR, using 25 µl Power SYBR Green PCR Master Mix (Applied Biosystems), 6.25 U MultiScribe Reverse Transcriptase (Applied Biosystems), 1.25 U RNase Inhibitor (Applied Biosystems), 0.2 µM of each primer (al1F 5′-CCATGTACATGGGCATGAGC-3′, al1R 5′-AGATACCCTCGGCCAACTCC-3′, al2F 5′-CCATCGGATCGGGGACGAAG-3′, al2R 5′-CCAGGAAGAACGTGGCTTCT-3′, tub-2F 5′-CCCGCGGTCTCAAGATGT-3′, tub-2R 5′-CGCTTGAAGAGCTCCTGGAT-3′) and 50 ng of RNA. Quantitative PCR analyses were performed using a 7500 Real Time PCR System (Applied Biosystems). The reaction included retrotranscription (30 min at 48°), denaturation (10 min at 95°), and 40 PCR cycles (15 sec at 95°, and 1 min at 60°). The results for each gene were normalized to the corresponding results obtained with *tub-2* to correct for sampling errors. Then, the results obtained with each sample were normalized to the RNA sample obtained from each mycelia kept in the dark.

### Survival of *Neurospora* conidia to UV radiation

Conidia from each *Neurospora* strain (400 µl, 10^5^ conidia/ml) were placed in a sterile Petri plate without the cover and exposed to UV radiation from a lamp (Philips TUV 15W/G15 T8 Longlife, emission of short-wave UV radiation with a peak at 253.7 nm) during different times. After UV exposure conidia were diluted and inoculated in plates with FGS agar that promotes colonial growth. All the manipulations were performed in dim light to prevent photoreactivation. The plates were incubated in the dark at 22°C during 2–3 days and colonies were counted. The percentage of survival to UV was obtained from the number of colonies after each UV treatment compared with the number of colonies in control plates with unirradiated conidia.

## Supporting Information

Table S1 Wild-type strains of *Neurospora* from Spain.
(XLS)

Table S2 A two-way analysis of variance (ANOVA) between *Neurospora* species, latitude, and carotenoid accumulation.
(DOCX)
