# Supplementary material for: A Relationship between Carotenoid Accumulation and the Distribution of Species of the Fungus Neurospora in Spain
Source: PLoS One. 2012 Mar 20;7(3):e33658. doi: 10.1371/journal.pone.0033658 (PMC3309001; doi:10.1371/journal.pone.0033658)
Supplement: Table S2 — A two-way analysis of variance (ANOVA) between Neurospora species, latitude, and carotenoid accumulation. (DOCX) [file pone.0033658.s002.docx]

**Table S2.** A two-way analysis of variance (ANOVA) between *Neurospora* species, latitude, and carotenoid accumulation.

|  | Low  Latitude (55) | Medium  Latitude (36) | High  Latitude (59) | Marginal means |
| --- | --- | --- | --- | --- |
| *N. crassa* (69) | 160 | 108 | NA | 141 |
| *N. tetrasperma* (17) | 116 | NA | 68 | 102 |
| *N. discreta* (63) | NA | 18 | 23 | 22 |
| Marginal means | 151 | 85 | 27 | 86 |

Average of carotenoids in mycelia exposed to light (µg/g dry mass). The independent factors are “Species” and “Latitude”, each with three levels: *N. crassa*, *N. tetrasperma* and *N. discreta* for factor “Species” and Low latitude (under 30º), Medium latitude (between 30º and 40º), and High latitude (over 40º) for factor “Latitude”. The number of isolates is shown in parenthesis. Means are rounded to the nearest integer. NA, not available. Umbalanced (empty cells) ANOVA was performed with PASW Statistics 18, release version 18.0.0 (www.spss.com).

*Neurospora* species show significant differences in carotenoid accumulation, and species from different latitudes show significant differences in carotenoid accumulation (p<0.001).
